# Selective degradation of the p53‐R175H oncogenic hotspot mutant by an RNA aptamer‐based PROTAC

**DOI:** 10.1002/ctm2.1191

**Published:** 2023-01-29

**Authors:** Lingping Kong, Fanlu Meng, Sijin Wu, Ping Zhou, Ruixin Ge, Min Liu, Linlin Zhang, Jun Zhou, Diansheng Zhong, Songbo Xie

**Affiliations:** ^1^ Department of Medical Oncology Tianjin Medical University General Hospital Tianjin China; ^2^ Laboratory of Molecular Modeling and Design State Key Laboratory of Molecular Reaction Dynamics Dalian Institute of Chemical Physics Chinese Academy of Sciences Dalian China; ^3^ Center for Cell Structure and Function Shandong Provincial Key Laboratory of Animal Resistance Biology Collaborative Innovation Center of Cell Biology in Universities of Shandong College of Life Sciences Shandong Normal University Jinan China; ^4^ Haihe Laboratory of Cell Ecosystem Tianjin China; ^5^ State Key Laboratory of Medicinal Chemical Biology College of Life Sciences Nankai University Tianjin China; ^6^ School of Life Sciences and Medicine Shandong University of Technology Zibo Shandong China

To the Editor:

*TP53* encodes the tumour suppressor protein p53, a master regulator of genomic integrity and cell survival, and is the most frequently mutated gene.^1^ p53 mutant proteins are stabilised and can acquire dominant‐negative or oncogenic gain‐of‐function activities, thereby promoting malignant transformation, metastasis and chemoresistance.^2^, ^3^ Proteolysis targeting chimeras (PROTACs) that hijack cellular ubiquitin‐proteasome machinery for targeted protein degradation have shown considerable promise in targeting previously undruggable proteins.^4^, ^5^ However, PROTACs targeting p53 mutants have not yet been reported, probably due to difficulty in identifying a suitable binder for these mutants. Some recent studies have revealed that aptamers can be exploited as ligands in place of standard peptides or small molecules for PROTACs.^6^, ^7^, ^8^ Here, using an aptamer‐based strategy, we developed the first selective hotspot p53 mutant PROTAC.

p53‐R175H is the most common p53 hotspot mutation.^9^ In this study, we used an RNA aptamer^10^ (hereafter named p53m‐RA) that selectively targets p53‐R175H as a binder for PROTAC development. First, we used streptavidin pulldown assays to confirm its binding specificity. Both p53m‐RA and N_3_‐p53m‐RA competitively abolished p53‐R175H pulldown from cell lysates with N_3_‐p53m‐RA‐biotin. By contrast, there was almost no pulldown of wild‐type p53 (p53‐WT) (Figure 1A). Structural analysis revealed that p53‐R175H displays a much narrower binding area for the DNA major groove than the wild‐type p53 (Figure 1B), but the newly formed groove between L2 and L3 facilitates the binding of p53m‐RA to p53‐R175H (Figure 1C). Moreover, the 5′ end of p53m‐RA was far away from the ligand binding sites (Figure 1D). Therefore, an alkynylated CRBN ligand (CRBNL), thalidomide‐O‐amido‐propargyl (Supporting Information Schemes), was connected to the 5′ end of N_3_‐p53m‐RA via a click reaction to generate the p53‐R175H degrader, dp53m‐RA (Figure 1E).

**FIGURE 1:**
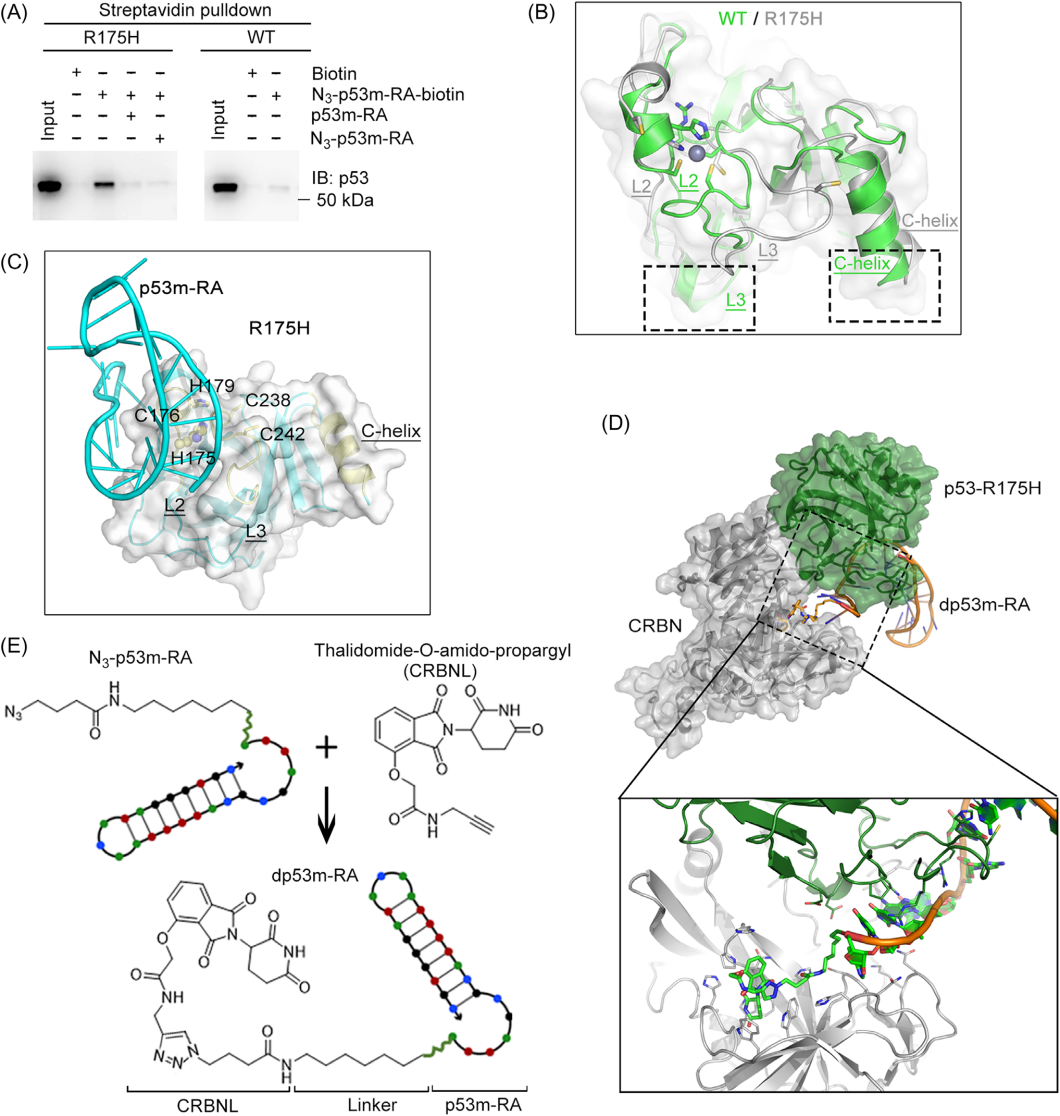
Harnessing a selective p53‐R175 aptamer as a binder for PROTAC development. (A) Streptavidin pulldown assay indicating that N_3_‐p53m‐RA‐biotin selectively binds to p53‐R175H but not p53‐WT. (B) Structural comparison of p53‐WT (green) and p53‐R175H (grey). The dashed boxes indicate the DNA binding areas. (C) Overall structure of p53‐R175H (grey) in complex with p53m‐RA (cyan). (D) A binding mode of dp53m‐RA (ball stick, yellow), p53‐R175H (PDB 1TSR, green) and CRBN (PDB 4CI3, grey) was simulated by protein–protein docking and molecular dynamics calculation. (E) Generation of the p53‐R175H degrader (dp53m‐RA) via a click reaction

Native polyacrylamide gel electrophoresis (PAGE) demonstrated a slight difference in dp53m‐RA migration relative to N_3_‐p53m‐RA, indicating successful conjugation (Figure 2A). Simulation analysis indicated that dp53m‐RA should actively bind p53‐R175H and CRBN simultaneously (Figure 1D). Indeed, dp53m‐RA retained its ability to compete with biotin‐p53m‐RA for binding to p53‐R175H, while CRBNL did not (Figure 2B), suggesting that the degrader could form a complex with CRBN and p53‐R175H. We next investigated its effects on p53‐R175H degradation. In p53‐R175H‐ or p53‐WT‐expressing H1299 cells (p53‐null), dp53m‐RA treatment destructed p53‐R175H, but spared p53‐WT in a dose‐dependent manner (Figure 2C). Time‐course experiments revealed that p53‐R175H was significantly degraded in 12 h (Figure 2D). To substantiate these findings, we treated SKBR3 (p53‐R175H), A549 (p53‐WT), and H1975 (p53‐R273H) cells with dp53m‐RA. As expected, dp53m‐RA treatment selectively degraded p53‐R175H, but not p53‐WT or p53‐R273H (Figure 2E). The half‐maximal degradation concentrations (DC_50_) for H1299‐p53‐R175H and SKBR3 cells were 1.06 and 1.28 µM, respectively (Figure 2F). When the effects of dp53m‐RA were examined on other hotspot mutations, only p53‐R175H was significantly degraded (Figure 2G). The expression levels of p53 downstream effectors, including MDM2, BAX, CDKN1A and PUMA, were elevated by dp53m‐RA treatment in H1299‐p53‐R175H and SKBR3 cells but not in H1299‐p53‐WT and A549 cells (Figure 2H). Furthermore, pretreatment of p53m‐RA compromised dp53m‐RA‐induced p53‐R175H degradation (Figure 2I). Moreover, the proteasome inhibitor MG132 completely blocked dp53m‐RA‐induced p53‐R175H degradation (Figure 2J), and dp53m‐RA treatment increased polyubiquitination of p53‐R175H over p53‐WT (Figure 2K). These findings indicate that dp53m‐RA is a selective p53‐R175H degrader.

**FIGURE 2:**
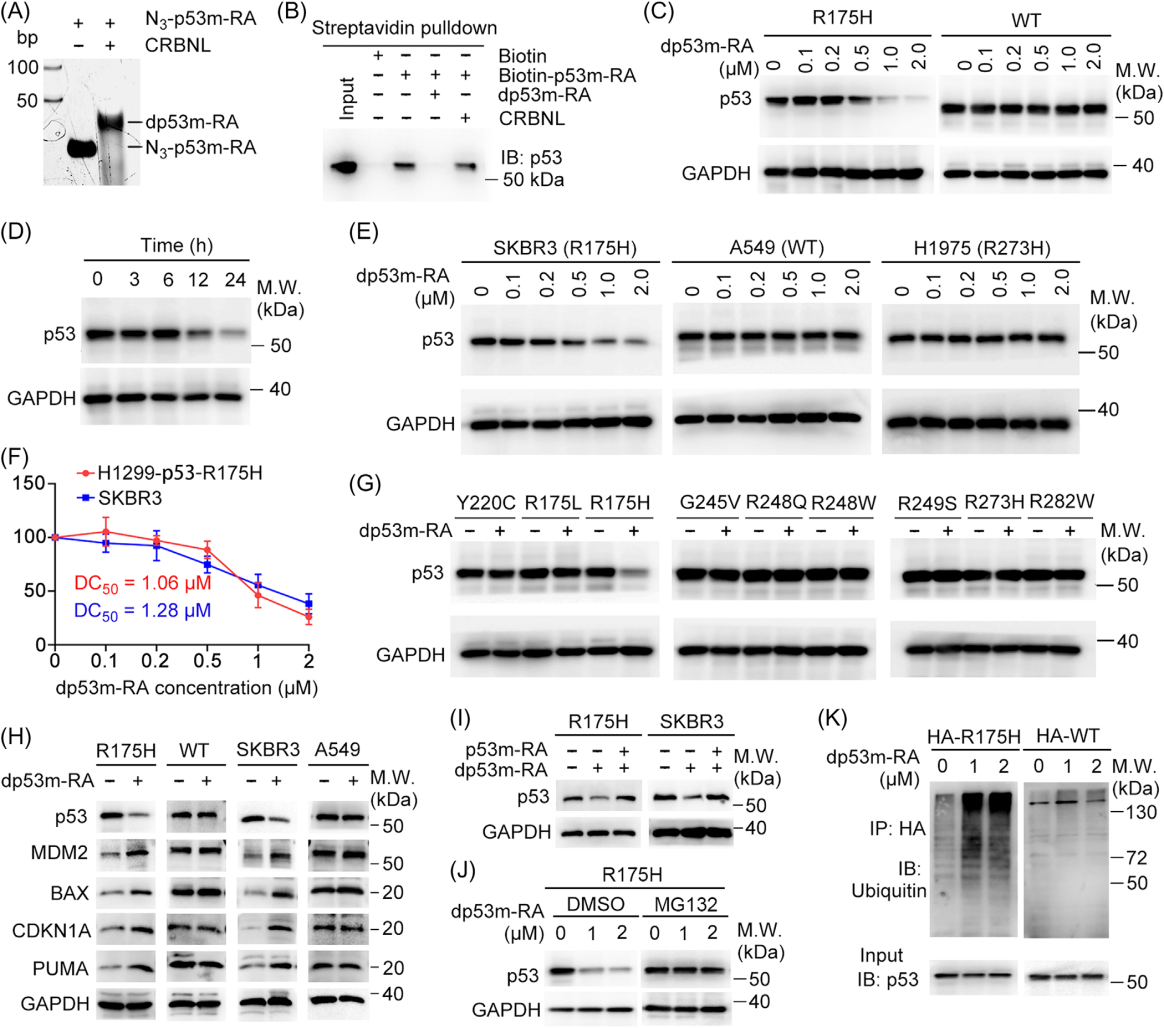
dp53m‐RA selectively degrades p53‐R175H in a ubiquitin‐proteasome‐dependent manner. (A) 20% Native PAGE analysis showing the conjugation of N3‐p53m‐RA to CRBNL. (B) Streptavidin pulldown assay indicating that dp53m‐RA competes with biotin‐p53m‐RA for binding to p53‐R175H. (C) H1299‐p53‐R175H or H2199‐p53‐WT cells were transfected with the indicated concentrations of dp53m‐RA, and lysates were subjected to immunoblotting. (D) H1299‐p53‐R175H cells were transfected with dp53m‐RA (1 µM) for the indicated times, and lysates were subjected to immunoblotting. (E) SKBR3, A549 and H1975 cells were treated with increasing concentrations of dp53m‐RA for 24 h, and lysates were subjected to immunoblotting. (F) The half‐maximal degradation concentrations (DC_50_) of dp53m‐RA on H1299‐p53‐R175H and SKBR3 cells were determined. (G) H1299 cells were transfected with the indicated p53 mutants and dp53m‐RA (1 µM) for 24 h, and lysates were subjected to immunoblotting. (H) Immunoblots for p53 downstream effectors in H1299‐p53‐R175H, H1299‐p53‐WT, SKBR3 and A549 cells. (I) H1299‐p53‐R175H and SKBR3 cells were pretreated with p53m‐RA (5 µM) followed by dp53m‐RA treatment (1 µM), and lysates were subjected to immunoblotting. (J) H1299‐p53‐R175H cells were transfected with dp53m‐RA (1 µM) in the presence or absence of MG132 (10 µM), and lysates were subjected to immunoblotting. (K) H1299 cells were co‐transfected with HA‐p53‐R175H and Myc‐ubiquitin plasmids, then treated with dp53m‐RA (1 µM). Cell lysates were subjected to immunoblotting.

We next examined the biological consequences of dp53m‐RA‐mediated p53‐R175H degradation in cancer cells. dp53m‐RA treatment dramatically inhibited the proliferation of p53‐R175H‐expressing H1299 cells and SKBR3 cells. By contrast, dp53m‐RA treatment did not affect the proliferation of p53‐WT‐expressing H1299 cells and A549 cells (Figure 3A,B). dp53m‐RA treatment consistently suppressed colony formation of p53‐R175H‐expressing cells, but not p53‐WT‐expressing cells (Figure 3C,D). However, p53m‐RA pretreatment did not diminish the anti‐tumour activities of dp53m‐RA (Figure S1A,B), probably due to the anti‐tumour activities of p53m‐RA per se at higher concentration. One advantage of dp53m‐RA over p53m‐RA is that it significantly lowers drug concentration because of its catalytic nature. Moreover, Transwell migration and wound‐healing assays revealed that dp53m‐RA significantly attenuated the migration of p53‐R175H‐expressing cells, but not p53‐WT‐expressing cells (Figure 3E–H). These results indicate that dp53m‐RA may have therapeutic potential in patients harbouring p53‐R175H mutant cancers.

**FIGURE 3:**
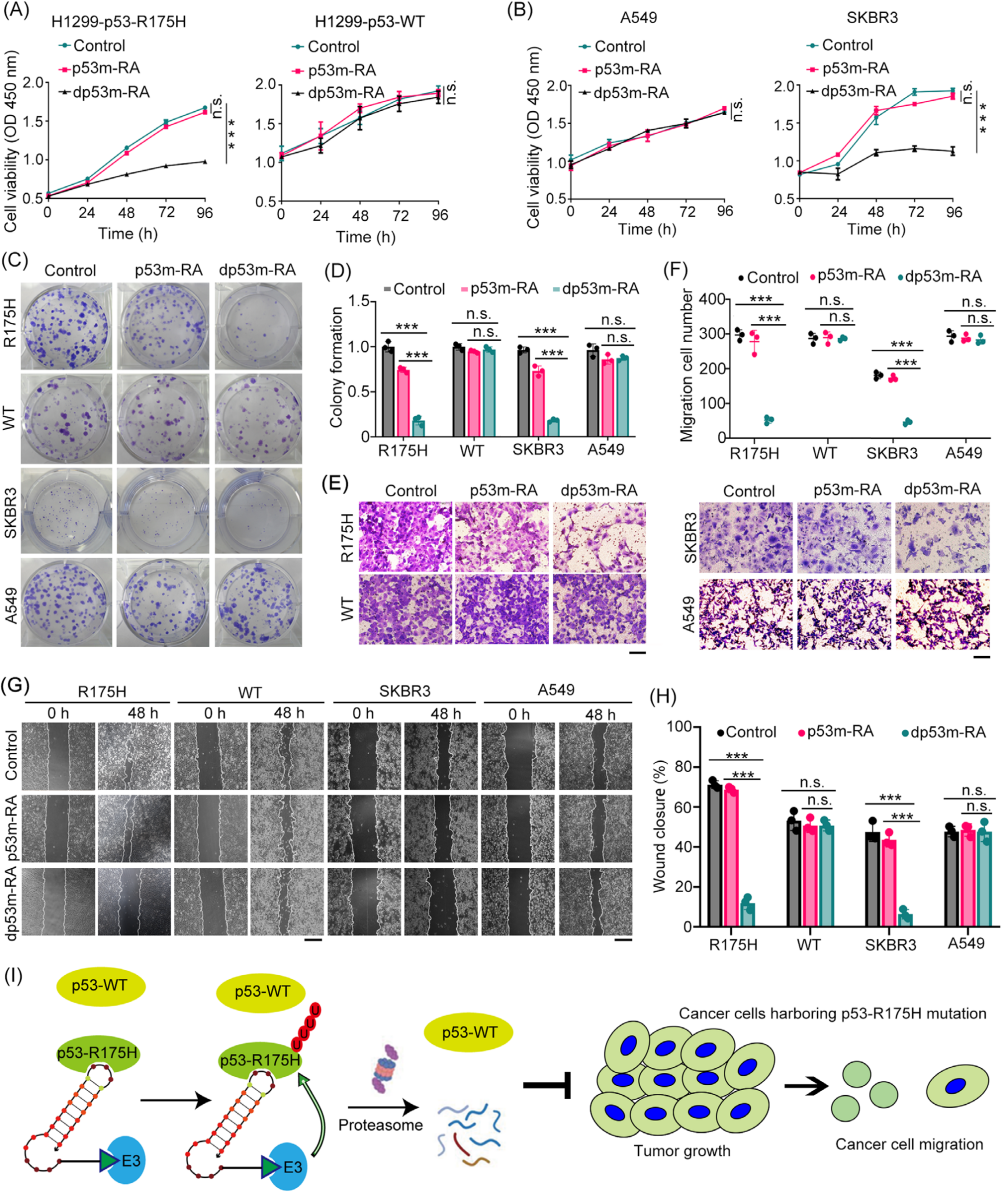
dp53m‐RA displays anti‐tumour activities in p53‐R175H‐expressing cancer cells. (A and B) H1299‐p53‐R175H, H1299‐p53‐WT cells, A549 and SKBR3 cells were treated with PBS, p53m‐RA (1 µM) or dp53m‐RA (1 µM) for the indicated times. Cell survival was determined with a CCK‐8 kit. (C and D) H1299‐p53‐R175H, H1299‐p53‐WT, SKBR3 and A549 cells were treated with PBS, p53m‐RA (1 µM) or dp53m‐RA (1 µM), and colony formation was determined and quantified. (E and F) Transwell images of H1299‐p53‐R175H, H1299‐p53‐WT, SKBR3 and A549 cells treated with PBS, p53m‐RA (1 µM) or dp53m‐RA (1 µM), and the migrated cells were quantified. Scale bar: 100 µm. (G and H) Wound healing images of H1299‐p53‐R175H, H1299‐p53‐WT, SKBR3 and A549 cells treated with PBS, p53m‐RA (1 µM) or dp53m‐RA (1 µM), and the wound closure was quantified. Scale bar: 500 µm. (I) Schematic showing the selective degradation of p53‐R175H protein by dp53m‐RA and its biological consequences. ***p < 0.001; Student's *t*‐test; n.s.: not significant

In summary, by harnessing an RNA aptamer specifically targeting p53‐R175H, the most common *TP53* mutation with dominant‐negative and oncogenic gain‐of‐function activities, as a binder in PROTAC design, we report the first selective mutant p53 degrader, dp53m‐RA. dp53m‐RA degraded p53‐R175H but not wild‐type p53 or other p53 mutants in a ubiquitin‐proteasome‐dependent manner. Importantly, dp53m‐RA inhibited the proliferation and migration of cancer cells specifically harbouring the p53‐R175H mutation (Figure 3I), suggesting its therapeutic potential for precision medicine.

## CONFLICT OF INTEREST

The authors declare they have no conflicts of interest.

## Supporting information

Supporting Information
