# Supplementary material for: Selective degradation of the p53‐R175H oncogenic hotspot mutant by an RNA aptamer‐based PROTAC
Source: Clin Transl Med. 2023 Jan 29;13(2):e1191. doi: 10.1002/ctm2.1191 (PMC9884801; doi:10.1002/ctm2.1191)
Supplement: Supplementary file 1 — Supporting Information [file CTM2-13-e1191-s001.docx]

**Supporting Information**

**Selective degradation of the p53-R175H oncogenic hotspot mutant by an RNA aptamer-based PROTAC**

Lingping Kong^1,^ ^†^, Fanlu Meng^1, †^, Sijin Wu^2, †^, Ping Zhou^3^, Ruixin Ge^3^, Min Liu^3,4^, Linlin Zhang^1^, Jun Zhou^3,4,5^, Diansheng Zhong^1,*^, Songbo Xie^3,4,6,*^

^1^Department of Medical Oncology, Tianjin Medical University General Hospital, Tianjin 300052, China

^2^Laboratory of Molecular Modeling and Design, State Key Laboratory of Molecular Reaction Dynamics, Dalian Institute of Chemical Physics, Chinese Academy of Sciences, Dalian 116024, China

^3^Center for Cell Structure and Function, Shandong Provincial Key Laboratory of Animal Resistance Biology, Collaborative Innovation Center of Cell Biology in Universities of Shandong, College of Life Sciences, Shandong Normal University, Jinan 250014, China

^4^Haihe Laboratory of Cell Ecosystem, Tianjin 300462, China

^5^State Key Laboratory of Medicinal Chemical Biology, College of Life Sciences, Nankai University, Tianjin 300071, China

^6^School of Life Sciences and Medicine, Shandong University of Technology, Zibo, Shandong 255000, China

**^†^These authors contributed equally to this work**

***Corresponding authors:** Diansheng Zhong (D.Z.); Songbo Xie (S.X.).

**Email:**  dzhong@tmu.edu.cn; xiesongbo@sdnu.edu.cn

**Materials and Methods**

**Antibodies, Chemicals, Aptamers and Plasmids**

Anti-p53 (Santa Cruz, Biotechnology, Inc., Dallas, TX, USA), anti-GAPDH (Proteintech, Rosemont, IL, USA), anti-HA (ABclonal Technology, Wuhan, China), anti-ubiquitin (Proteintech, Wuhan, China), streptavidin agarose beads (Thermo Fisher Scientific, Waltham, MA, USA), anti-HA magnetic beads (Bimake, Houston, TX, USA), and MG132 (Selleck Chemicals, Radnor, PA, USA) were purchased from the indicated sources. Horseradish peroxidase-conjugated secondary antibodies were obtained from ABclonal Technology. The RNA aptamer (5’-AUUAGCGCAUUUUAACAUAGGGUGC-3’) and its modified versions were synthesized by Beijing Genomics Institute (Beijing, China). N_3_-p53m-RA was synthesized by incorporating an azide group onto the 5´ end of the aptamer through a 5´ amino modifier C6. The pCDNA3.1-HA-p53 plasmid was from the Public Protein/Plasmid Library (Nanjing, China), and the plasmids expressing site-directed mutants of p53 were constructed by PCR.

**dp53-RA Generation**

To generate dp53m-RA, thalidomide-O-amido-propargyl was incubated with N_3_-p53m-RA in the reaction buffer (10 mM Tris-HCl, 10 mM CuSO_4_, 10 mM sodium erythorbate) at 37°C for 4 hours. The reaction mixtures were purified by 3K ultrafiltration (Millipore, Burlington, MA, USA) to remove excessive CRBNL.

**Cell Culture and Treatment**

H1299, SKBR3, A549, and H1975 cells were obtained from the American Type Culture Collection. Cells were cultured in RPMI 1640 or DMEM (Thermo Fisher Scientific) supplemented with 10% fetal bovine serum. dp53m-RA and plasmids were transfected into cells with Lipofectamine 3000 (Thermo Fisher Scientific).

**Native DNA Polyacrylamide Gel Electrophoresis (PAGE)**

p53m-RA and dp53m-RA were separated by 20% native polyacrylamide gel electrophoresis (PAGE), followed by incubation in 0.2% EtBr solution in 1 x Tris-acetate-EDTA (TAE) buffer and staining with a GelRed solution (UE Everbright, Suzhou, China). Gels were imaged by UV illumination with a ChemiDoc Imaging System (Bio-Rad, Hercules, CA, USA).

**Immunoblotting**

Proteins were electrophoresed by 10% SDS-PAGE electrophoresis and transferred onto polyvinylidene difluoride membranes (Millipore, Burlington, MA, USA). Membranes were blocked with 5% fat-free milk in Tris-buffered saline containing 0.1% Tween 20, followed by incubation with primary antibodies at 4°C overnight and secondary antibodies for 1 hour at room temperature. The targets were detected with an enhanced chemiluminescence substrate kit (Millipore).

**Streptavidin Pulldown Assays**

Cell lysates from H1299 cells ectopically expressing HA-p53-R175H or HA-p53-WT were incubated with biotin-p53m-RA (10 μM) at 4°C for 4 hours. 30 μL of streptavidin agarose beads were then added into the lysates and incubated at 4°C overnight. The beads were washed 6 times, boiled in 2 x SDS loading buffer, separated by 10% SDS-PAGE electrophoresis, and immunoblotted.

**Immunoprecipitation**

H1299 cells were co-transfected with HA-p53-R175H or HA-p53-WT and Myc-ubiquitin plasmids for 24 hours, followed by transfecting with dp53m-RA in the presence of MG132 for another 12 hours. Cell lysates were incubated with anti-HA magnetic beads at 4°C overnight. Immunoprecipitates were resolved by 10% SDS-PAGE and immunoblotted with ubiquitin antibodies.

**Cell Proliferation and Colony Formation Assays**

For cell proliferation assays, cells were seeded into 96-well plates, transfected with control, p53m-RA, or dp53m-RA for the indicated times, followed by addition of CCK-8 kit substrate (Solarbio, Beijing, China) for cell survival determination. For colony formation assays, cells (1000/well) in a 6-well plate were transfected with control, p53m-RA, or dp53m-RA every two days. Two to three weeks later, the cells were fixed with 4% paraformaldehyde and stained with 0.4% crystal violet in 20% ethanol. The colonies were imaged and clones quantified using ImageJ software.

**Cell Migration Assays**

Cell migration assays were conducted as described previously.^1^ Briefly, for transwell assays, cells pre-treated with control, p53m-RA, or dp53m-RA were resuspended in serum-free medium and seeded into transwell chambers. After incubation for 24 hours, cells in the upper chamber were removed, and cells in the lower chambers were fixed with paraformaldehyde and stained with crystal violet solution. Photographs were taken with an inverted microscope (Olympus, Tokyo, Japan). For wound healing assays, confluent cells in 24-well plates were starved overnight and scratched with a 10 μL tip. After wash with PBS three times, cells were with control, p53m-RA, or dp53m-RA for the indicated time, and the wound was imaged with an inverted microscope (Olympus).

**Structural Simulation**

To study the structural changes of p53-R175H and the selectivity mechanism of p53m-RA, the crystal structure of p53 (1TSR, chain B) was taken to do the mutation at R175 with Swiss-PDB viewer and the following 1 μs MD simulation to obtain the equilibrium state of p53-R175H. The online server RNAComposer was used to build the 3D structure of p53m-RA. The complex structure of p53-R175H, p53m-RA and CRBN (4CI3, chain B) was built with ZDOCK and RosettaDock, followed with 500 ns MD simulation. And 50 ns MD simulation was conducted to sample the linker structure of dp53m-RA to build the complete structure. Another 500 ns MD simulation of dp53m-RA, p53-R175H and CRBN complex was calculated to study the binding capacity of dp53m-RA as a PROTAC.

**Statistical Analysis**

GraphPad Prism v8.0 (GraphPad Software, La Jolla, CA, USA) was used for statistical analyses. Significant differences were determined using Student’s *t*-test, with *P*-values less than 0.05 considered statistically significant.

**Supplementary Figure 1**

**
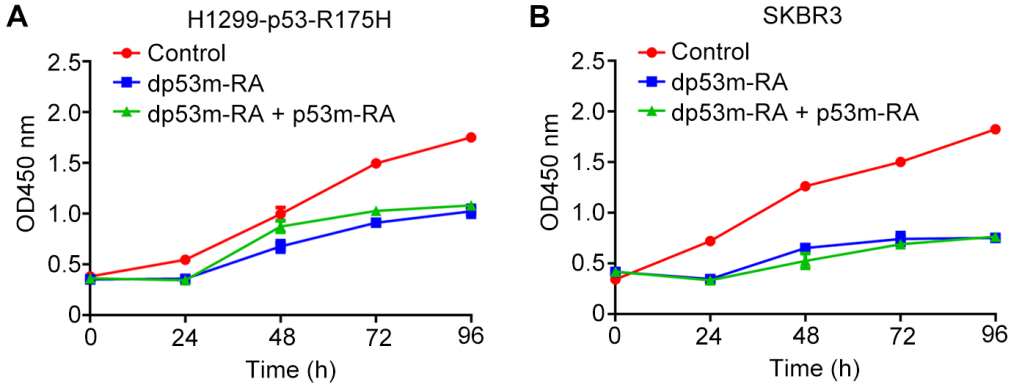
**

**Fig S1.** H1299-p53-R175H (A) and SKBR3 (B) cells were treated with PBS, dp53m-RA (1 μM), or pretreated with p53m-RA (5 μM) followed by dp53m-RA treatment (1 μM) and incubated for the indicated time points. The viable cells were determined by a CCK-8 kit.

**Supporting Schemes**

**Synthesis of the CRBN ligand g (thalidomide-O-amido-propargyl)**

A mixture of 4-hydroxyisobenzofuran **a** (3 mmol), 3-aminopiperidine **b** (3 mmol) in anhydrous toluene (20 mL), and triethylamine (TEA, 3.36 mmol) was stirred in a round bottom flask for 12 hours at 110°C. The mixture was cooled to room temperature and filtered. The obtained filtered cake was washed with EtOAc/PE (3:10) solution for 10 minutes. The residue was then filtered and washed with petroleum ether (PE). The product **c** was purified by flash chromatography.

TEA (1.2 mmol) was added to 3-aminopropyne **e** (1 mmol) in dichloromethane (DCM, 10 mL) and the mixture stirred at 0°C. Subsequently, bromoacetyl bromide **d** in DCM was added dropwise into the reaction solution. The mixture was stirred at room temperature for at least 2 hours. Finally, the residue was washed with PE twice, and the product **f** (white solid) was purified by flash chromatography (EtOAc/PE).

A mixture of the product **c**, **d** (2 mmol) in DMF, and K_2_CO_3_ (6 mmol) was reacted at room temperature for 2 hours. The mixture was diluted with EtOAc and washed once with water then twice with saline. The organic layer was dried over sodium sulfate, filtered, and concentrated under reduced pressure. A cream-colored solid product **g** (thalidomide-O-amido-propargyl) was obtained by purification with column chromatography (EtOH/DCM).

^1^H N**MR Spectrum of thalidomide-O-amido-propargyl.**


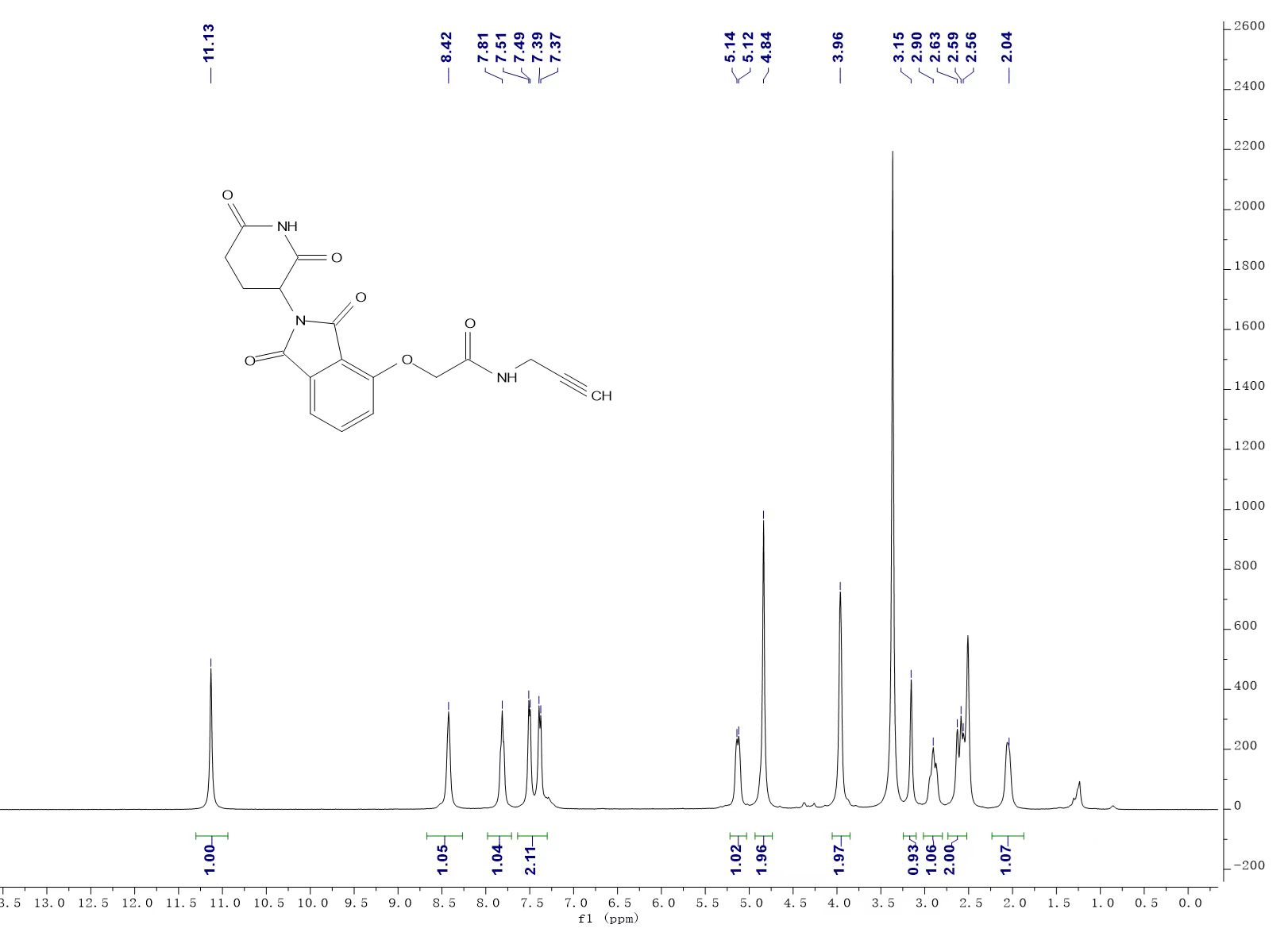
2-((2-(2,6-dioxopiperidin-3-yl)-1,3-dioxoisoindolin-4-yl)oxy)-N-(prop-2-yn-1-yl)acetamide (**g**). 1H NMR (400 MHz, CDCl3): δ ppm 7.75-7.68 (m, 2H), 7.53-7.51 (d, J = 8 Hz, 1H), 7.23-7.21 (d, J = 8 Hz, 1H), 6.69 (br, 1H), 5.17-5.13 (dd, J = 12 Hz, J = 4 Hz, 1H), 4.69 (s, 2H), 4.56-4.47 (m, 2H), 3.02-2.78 (m, 3H), 2.24 (br, 1H), 2.16 (br, 1H).

^13^C N**MR Spectrum of thalidomide-O-amido-propargyl.**


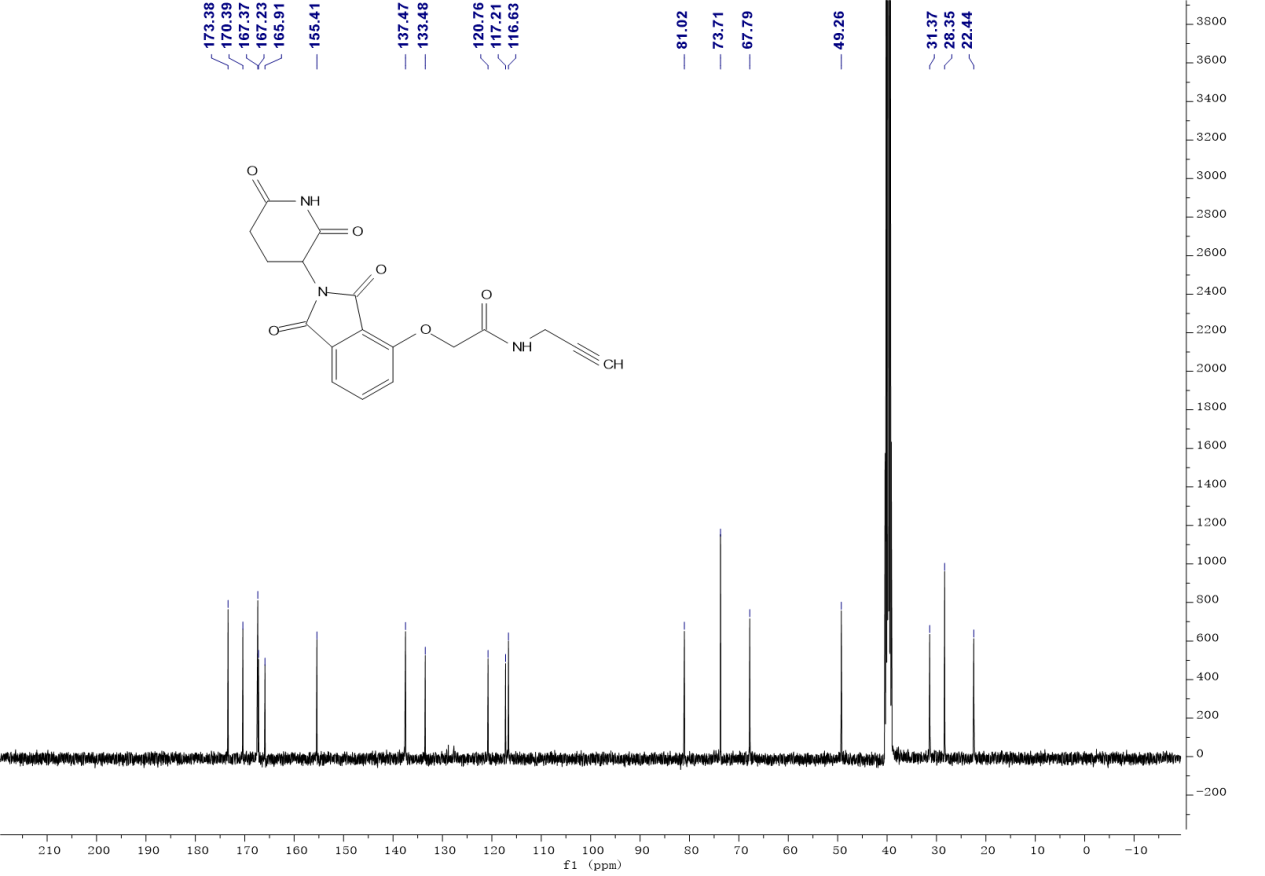


**Mass spectrometry of thalidomide-O-amido-propargyl.**


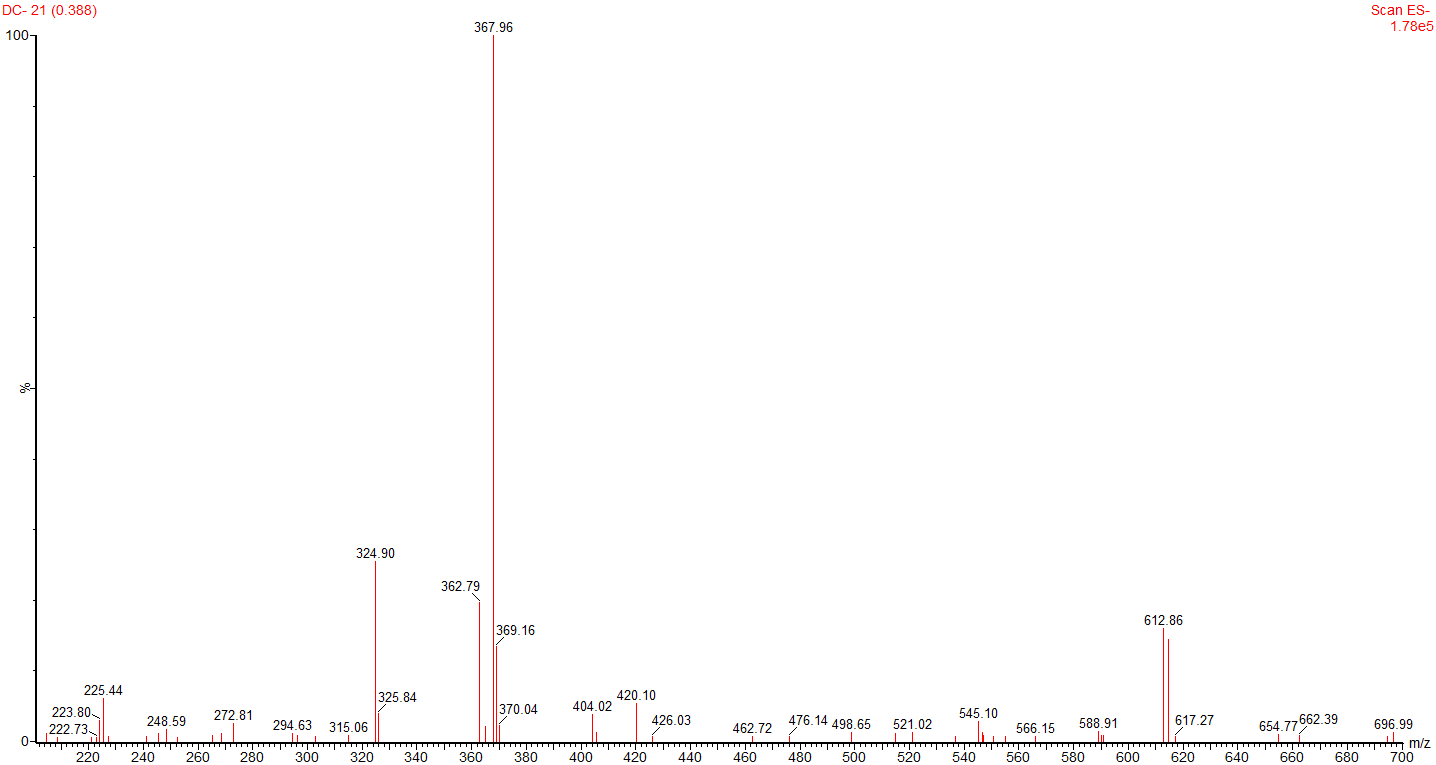


**LC/MS of dp53m-RA**

**References**

1. Yang Y; Chen M; Li J, et al. A cilium-independent role for intraflagellar transport 88 in regulating angiogenesis. *Sci Bull.* **2021;** 66: 727-739.
